# A New Approach for Estimating Dissolved Oxygen Based on a High-Accuracy Surface Modeling Method

**DOI:** 10.3390/s21123954

**Published:** 2021-06-08

**Authors:** Na Zhao, Zemeng Fan, Miaomiao Zhao

**Affiliations:** 1 State Key Laboratory of Resources and Environmental Information System, Institute of Geographic Sciences and Natural Resources Research, Chinese Academy of Sciences, Beijing 100101, China; zhaomm@lreis.ac.cn; 2 College of Resources and Environment, University of Chinese Academy of Sciences, Beijing 100101, China; 3 Jiangsu Center for Collaborative Innovation in Geographic Information Resource Development and Application, Nanjing 210023, China

**Keywords:** surface modeling, dissolved oxygen, spatial estimation

## Abstract

Dissolved oxygen (DO) is a direct indicator of water pollution and an important water quality parameter that affects aquatic life. Based on the fundamental theorem of surfaces in differential geometry, the present study proposes a new modeling approach to estimate DO concentrations with high accuracy by assessing the spatial correlation and heterogeneity of DO with respect to explanatory variables. Specifically, a regularization penalty term is integrated into the high-accuracy surface modeling (HASM) method by applying geographically weighted regression (GWR) with some covariates. A modified version of HASM, namely HASM_MOD, is illustrated through a case study of Poyang Lake, China, by comparing the results of HASM, a support vector machine (SVM), and cokriging. The results indicate that HASM_MOD yields the best performance, with a mean absolute error (MAE) that is 38%, 45%, and 42% lower than those of HASM, the SVM, and cokriging, respectively, by using the cross-validation method. The introduction of a regularization penalty term by using GWR with respect to covariates can effectively improve the quality of the DO estimates. The results also suggest that HASM_MOD is able to effectively estimate nonlinear and nonstationary time series and outperforms three other methods using cross-validation, with a root-mean-square error (RMSE) of 0.20 mg/L and R^2^ of 0.93 for the two study sites (Sanshan and Outlet_A stations). The proposed method, HASM_MOD, provides a new way to estimate the DO concentration with high accuracy.

## 1. Introduction

As a health indicator for water bodies, the dissolved oxygen (DO) concentration plays important roles in maintaining microbial diversity and various ecosystem and biogeochemical processes in lake ecosystems [1,2,3]. Sufficient levels of DO in water are essential for the survival of various aquatic organisms, such as algae, zooplankton, and aquatic plants. Generally, the DO concentration in a healthy water body ranges from 8 to 12 mg/L, and concentrations below 8 mg/L can adversely affect the survival of aquatic species [4,5]. Studies have shown that global climate change and extensive human activities can rapidly reduce DO concentrations, leading to increased hypoxia, especially in coastal and estuarine environments [6,7]. According to the National Oceanic and Atmospheric Administration (NOAA), any persistent DO levels below 5.0 mg/L are considered unhealthy, and levels below 2 mg/L are extremely hazardous to marine ecosystems [6]. Reliable estimates of DO concentrations enable us to identify future contaminant problems and provide a basis for taking effective countermeasures to prevent water pollution.

Although in situ observations with sensors provide highly accurate measurements of DO concentrations, this approach is intensive and only gives point measurements. Studies have shown that no single identified sensor can be applied with high confidence to optimally measure DO concentrations [8]. An improved understanding of water quality can be obtained by integrating remote sensing technology, site observations, and numerical models. Recently, various approaches have been proposed for the estimation of DO concentrations. The main prediction methods include various deterministic hydrological models, statistical models, and machine learning methods [9,10,11,12,13]. Hydrological models can provide physical DO concentration estimates but are characterized by large computational capacities and high uncertainty associated with the determination of parameters and relevant physical processes [14]. The statistical method is a simple approach used to generate spatial DO concentration fields. Harvey et al. [15] established a regression model to predict the monthly water temperature and DO level. Stajkowski used an autoregressive integrated moving average (ARIMA) model to estimate water quality parameters and demonstrated its capability in DO concentration prediction [16]. In many statistical methods, the geostatistical method known as kriging has been widely applied, and cokriging is an extension of kriging used when estimating one variable from other variables [17,18]. The literature shows that cokriging has been successfully used for the prediction and estimation of groundwater quality parameters [19]. However, statistical methods generally depend on datasets with linear relationships and fail to describe nonlinear characteristics. Machine learning methods can be applied to address nonlinear problems. Support vector machines (SVMs) and artificial neural networks (ANNs) are typical models that are commonly adopted by researchers in the prediction of water quality and have displayed good performance in modeling DO concentrations [20,21,22,23,24]. ANNs are well-suited methods with self-adaptability, self-organization, and error tolerance for nonlinear simulation, but these methods have limitations due to their complex structures that require a great amount of training data. SVMs are new machine-learning technologies based on statistical theory and derived from instruction risk minimization that can enhance the generalization ability and minimize the upper limit of generalization error [25]. However, as indicated in other studies [26,27], machine learning methods are usually applied to establish global numeric relationships among datasets and ignore geographical relations; notably, environmental parameters are often spatially correlated, and the relationships among variables varying in space are important.

In recent years, a partial differential equation (PDE)-based approach, the high-accuracy surface modeling (HASM) method, was proposed based on the fundamental theorem of surfaces in differential geometry by using in situ measurements [28]. This method is effective in simulating elevations, climate variables, soil properties, ecological diversity, and other ecological variables. Researchers have found that HASM generally performs better than other classic interpolation methods, such as kriging, inverse distance weighting (IDW), and the spline method [29,30,31,32,33,34]. The good performance of HASM is due to its complete theoretical basis and the constraint conditions established using sampling information. One of the advantages of HASM is its extensibility, which allows it to simulate different environmental variables based on the characteristics of the variables and the corresponding a priori knowledge. The performance of HASM in estimating the DO concentration, however, has not been investigated until now. This approach is expected to provide an effective way to improve the accuracy of the spatial estimation of DO levels.

The objective of this study is to propose a new approach for estimating the spatial distribution of DO concentrations in Poyang Lake, China. The new method, termed HASM_MOD, was developed based on HASM and the characteristics of DO by modifying the main HASM equation. The performance of HASM_MOD was investigated by comparing it with HASM, cokriging, and SVM using a cross-validation method. This is the first study in the literature that attempts to apply HASM and HASM_MOD to simulate DO concentrations. The rest of this study is organized as follows: In Section 2, the materials are explained, including the details of the study area and datasets used in the analysis. The methods are presented in Section 3, and the results are shown in Section 4. Discussions and conclusions are given in Section 5 and Section 6, respectively.

## 2. Study Area and Data

**Figure 1:**
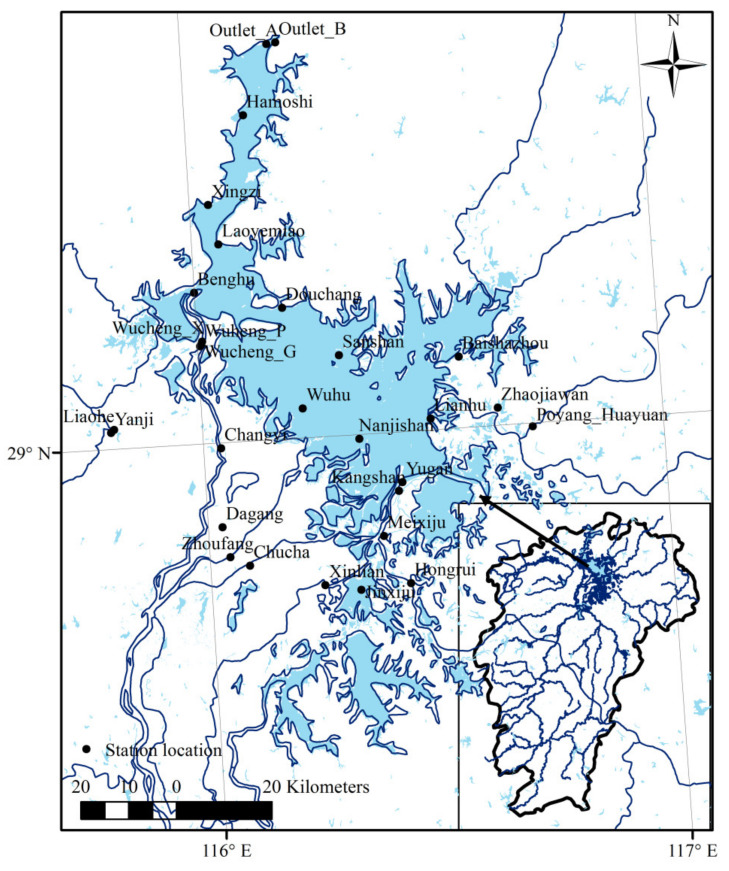
Locations of DO measurements and the Poyang Lake boundary.

Poyang Lake, the largest freshwater lake in China, is located downstream of the Yangtze River in Jiangxi Province and freely connected to the longest river in China, the Yangtze River (Figure 1). The lake has a southeast monsoonal climate with an annual mean air temperature of 16–18 °C and an average annual precipitation of 1340–1780 mm. The elevation of the Poyang Lake area gradually increases from north to south and west to east. Poyang Lake is considered a vital resource not only for the local population but also for the Yangtze valley and global ecology. The lake routinely fluctuates in volume between the winter and summer seasons. The surface area of Poyang Lake changes greatly with season, covering more than 4000 km^2^ in the wet season (June, July, August, and September) and a much smaller area in the comparatively dry season (lasting from October to March in the following year) [35]. The lake water level is mainly regulated by the Yangtze River and the “five rivers”, namely the Gan River, Fu River, Xin River, Rao River, and Xiu River, with a range of water levels spanning more than 10 m. The water level rises first due to the inflows from the five rivers from April to June and then due to the backflow of the Yangtze River from July to September; the water levels then gradually fall after October, with the decreasing trend lasting for approximately half a year [36]. Influenced by the topographic characteristics of Poyang Lake and the variations in the runoff of the five rivers, the surface area and water level of Poyang Lake vary greatly with the season. In addition, it has been reported that polluted river sections are mainly located in the Gan River, Fu River, Rao River, and Xiu River. This study performed an analysis based on the observations obtained at stations located in Poyang Lake and the surrounding inflowing rivers.

The data used in this work consist of daily measurements of DO (mg/L), pH, water temperature (TE, Co), and electrical conductivity (EC, μS/cm) from 30 monitoring stations equipped with different sensors. Fourteen stations were located in the five main inflowing rivers, and the corresponding data were included in the calculation process to improve the estimations. DO concentrations were measured using a Hatch luminescent DO sensor and were transferred via SODA (SODA open data autonomy) telemetry to a central database. The water quality parameters, which were collected by monitoring centers, were quality controlled and then sent to the server through GPRS.

Previous studies predicted DO levels using several environmental and meteorological variables as inputs [16,24,37,38]. However, the conclusions were inconsistent due to variations in the study area and spatiotemporal scales. For example, Heddam [39] found that high accuracy can be obtained for DO estimations by using only water temperature (WT) as the input variable. Researchers have also used other variables, such as pH, chlorophyll-a (Chl-a), and humidity. Rankovic et al. [20] indicated that WT and pH have the greatest effects on the DO concentration. Najah et al. [40] demonstrated that water pH, as an input variable, had a very limited influence on the performance of DO prediction models in Johor, Malaysia. Li et al. [41] showed that changes in DO are mainly affected by meteorological variables, such as atmospheric temperature (AT), atmospheric pressure (AP), and precipitation (Pre). Researchers also found that among the considered variables, WT has the highest correlation with DO [8]. In this study, the correlations among environmental factors and DO were estimated by using Spearman’s rank method. The candidate factors included WT, pH, Chl-a, EC, AT, AP, and precipitation (Pre). The Chl-a concentration was derived based on Landsat-8 OLI using the following formula [42]:

$$Chl-a=124.3\times {\left(B4\right)}^{2}+15.28\times B4+0.914,R^{2}=0.846$$

**Figure 2:**
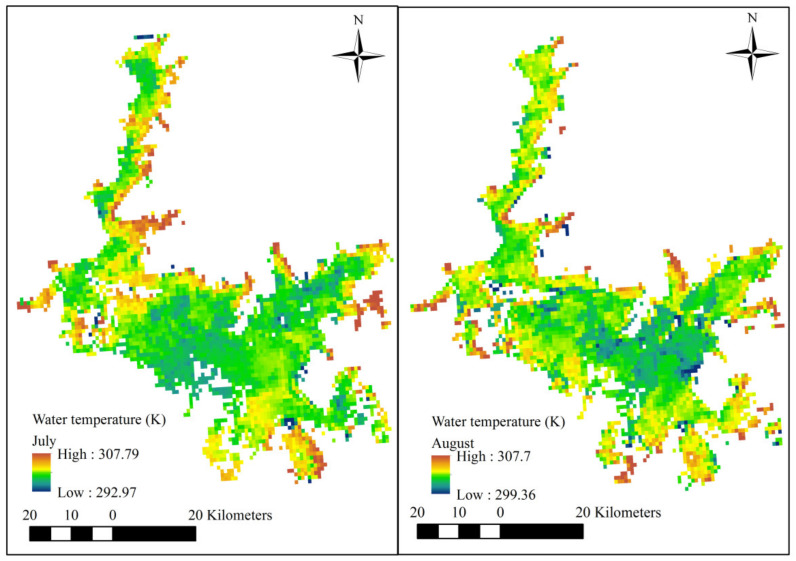
The Poyang Lake boundary obtained using satellite-derived lake water surface temperatures in July and August.

The surface water temperature can be retrieved with a satellite-based remote sensor that detects thermal radiation (3–5 and 8–14 µm wavebands) emitted from the upper 0.1 mm of the water surface [43,44]. Since the water volume of Poyang Lake fluctuates seasonally, thermal remote sensing of WTs can be applied to provide the boundary of the water body in different months. The satellite-derived lake water surface temperatures used in this study were provided by the European Space Agency (ESA) Lakes Climate Change Initiative (Lakes-cci) project [45]. Figure 2 displays the water body boundary in the summer months (taking July and August as two examples).

## 3. Methods

### 3.1. HASM

According to the theory of the differential geometry of surfaces, a surface can be uniquely determined by its first and second fundamental coefficients [46,47]. The first fundamental coefficients of a surface, z=f(x,y), are expressed as

$$E=1+f_{x}^{2},F=f_{x}\cdot f_{y},G=1+f_{y}^{2},$$

and they reflect geodesic curvature, curve length, and other intrinsic geometric information. The second fundamental coefficients characterize the local structures of the surface and are expressed as [48,49]

$$L=\frac{f_{xx}}{\sqrt{1+f_{x}^{2}+f_{y}^{2}}},\;M=\frac{f_{xy}}{\sqrt{1+f_{x}^{2}+f_{y}^{2}}},\;\mathrm{and}\;N=\frac{f_{yy}}{\sqrt{1+f_{x}^{2}+f_{y}^{2}}}.$$

Based on the theorem of surfaces, a surface can be obtained by solving the Gaussian equation set based on the condition that the first and second fundamental coefficients satisfy this equation set [47]. The assumption of HASM is that the spatial distribution of the predictor is deemed a surface that can be obtained by solving Gaussian equations. Therefore, the main equations of HASM are the following Gaussian equations:

(1) $$\left\{\begin{array}{c} f_{xx}=\Gamma_{11}^{1}f_{x}+\Gamma_{11}^{2}f_{y}+\frac{L}{\sqrt{E+G-1}}\\ f_{yy}=\Gamma_{22}^{1}f_{x}+\Gamma_{22}^{2}f_{y}+\frac{N}{\sqrt{E+G-1}}\\ f_{xy}=\Gamma_{12}^{1}f_{x}+\Gamma_{12}^{2}f_{y}+\frac{M}{\sqrt{E+G-1}} \end{array}\right.,$$

where $f_{x}$, $f_{y}$, $f_{xx}$, and $f_{yy}$ are the first and second partial derivatives of the graph z=f(x,y) with respect to the x and y directions, respectively.

Let $\left\{\left(x_{i},y_{j}\right)|0\leq i\leq I+1,0\leq j\leq J+1\right\}$ be the calculation grids and h be the grid spacing. The finite discrete schemes of $f_{x}$, $f_{xx}$, $f_{y}$ and $f_{yy}$ can be given as

$${\left(f_{x}\right)}_{\left(i,j\right)}\approx \left\{\begin{array}{c} \frac{f_{1,j}-f_{0,j}}{h}\;i=0\\ \frac{f_{i+1,j}-f_{i-1,j}}{2h}i=1,\cdots ,I,\\ \frac{f_{I+1,j}-f_{I,j}}{h}i=I+1 \end{array}\right.\;{\left(f_{xx}\right)}_{\left(i,j\right)}\approx \left\{\begin{array}{c} \frac{f_{0,j}-2f_{1,j}+f_{2,j}}{h^{2}}\;i=0\\ \frac{f_{i-1,j}-2f_{i,j}+f_{i+1,j}}{h^{2}}i=1,\cdots ,I\\ \frac{f_{I+1,j}-2f_{I,j}+f_{I-1,j}}{h^{2}}\;i=I+1 \end{array}\right.\;{\left(f_{y}\right)}_{\left(i,j\right)}\approx \left\{\begin{array}{c} \frac{f_{i,1}-f_{i,0}}{h}\;j=0\\ \frac{f_{i,j+1}-f_{i,j-1}}{2h}j=1,\cdots ,J,\\ \frac{f_{i,J+1}-f_{i,J}}{h}\;j=J+1 \end{array}\right.\;{\left(f_{yy}\right)}_{\left(i,j\right)}\approx \left\{\begin{array}{c} \frac{f_{i,0}-2f_{i,1}+f_{i,2}}{h^{2}}\;j=0\\ \frac{f_{i,j-1}-2f_{i,j}+f_{i,j+1}}{h^{2}}j=1,\cdots ,J\\ \frac{f_{i,J+1}-2f_{i,J}+f_{i,J-1}}{h^{2}}j=J+1 \end{array}\right.$$

The first partial derivatives $f_{x}$ and $f_{y}$ represent the variations in the predictor in the x and y directions, respectively, and the second partial derivatives $f_{xx}$ and $f_{yy}$ denote the slope and direction of the variations in the predictor in the x and y directions, respectively. $f_{xy}$ is the mixed partial derivative and represents the cross-slope of the change in the x and y directions.

$${\left(f_{xy}\right)}_{\left(i,j\right)}\approx \left\{\begin{array}{c} \frac{f_{1,1}-f_{1,0}-f_{0,1}+f_{0,0}}{h^{2}}\;i=0,j=0\\ \frac{f_{1,J+1}-f_{1,J}-f_{0,J+1}+f_{0,J}}{h^{2}}\;i=0,j=J+1\\ \frac{f_{1,j+1}-f_{0,j+1}-f_{1,j-1}+f_{0,j-1}}{2h^{2}}\;i=0,j=1,\cdots ,J\\ \frac{f_{I+1,1}-f_{I,0}-f_{I,1}+f_{I+1,0}}{h^{2}}\;i=I+1,j=0\\ \frac{f_{I,J}-f_{I+1,J}-f_{I,J+1}+f_{I+1,J+1}}{h^{2}}\;i=I+1,j=J+1\\ \frac{f_{I+1,j+1}-f_{I,j+1}-f_{I+1,j-1}+f_{I,j-1}}{2h^{2}}\;i=I+1,j=1,\cdots ,J\\ \frac{f_{i+1,1}-f_{i+1,0}-f_{i-1,1}+f_{i-1,0}}{2h^{2}}\;i=1,\cdots ,I,j=0\\ \frac{f_{i+1,J+1}-f_{i+1,J}-f_{i-1,J+1}+f_{i-1,J}}{2h^{2}}\;i=1,\cdots ,I,j=J+1\\ \frac{f_{i+1,j}-f_{i+1,j-1}-f_{i-1,j+1}-2f_{i,j}+f_{i,j-1}+f_{i,j+1}+f_{i-1,j}}{2h^{2}}\;i=1,\cdots ,I,j=1,\cdots ,J. \end{array}\right.$$

The Christoffel symbols ($\Gamma_{11}^{1},\Gamma_{11}^{2},\Gamma_{22}^{1},\Gamma_{22}^{2}$) are shorthand notations for various functions associated with second derivatives [49] and here depend only on the first fundamental coefficients and their derivatives:

$$\begin{array}{l} \Gamma_{11}^{1}=\frac{1}{2}(GE_{x}-2FF_{x}+FE_{y}){\left(EG-F^{2}\right)}^{-1},\;\Gamma_{11}^{2}=\frac{1}{2}(EF_{x}-2EE_{y}+FE_{x}){\left(EG-F^{2}\right)}^{-1},\\ \Gamma_{22}^{1}=\frac{1}{2}(2GF_{y}-2GG_{x}+FG_{y}){\left(EG-F^{2}\right)}^{-1},\;\Gamma_{22}^{2}=\frac{1}{2}(EG_{y}-2FF_{y}+FG_{x}){\left(EG-F^{2}\right)}^{-1}\\ \Gamma_{12}^{1}=\frac{1}{2}(GE_{y}-FG_{x}){\left(EG-F^{2}\right)}^{-1},\;\Gamma_{12}^{2}=\frac{1}{2}(EG_{x}-FE_{y}){\left(EG-F^{2}\right)}^{-1}. \end{array}$$

The differential Equation (1) can be converted into the following finite difference equations by applying Taylor expansions with finite difference schemes:

(2) $$\left\{\begin{array}{c} \frac{f_{i+1,j}^{n+1}-2f_{i,j}^{n+1}+f_{i-1,j}^{n+1}}{h^{2}}={\left(\Gamma_{11}^{1}\right)}_{i,j}^{n}\frac{f_{i+1,j}^{n}-f_{i-1,j}^{n}}{2h}+{\left(\Gamma_{11}^{2}\right)}_{i,j}^{n}\frac{f_{i,j+1}^{n}-f_{i,j-1}^{n}}{2h}+\frac{L_{i,j}^{n}}{\sqrt{E_{i,j}^{n}+G_{i,j}^{n}-1}}\\ \frac{f_{i,j+1}^{n+1}-2f_{i,j}^{n+1}+f_{i,j-1}^{n+1}}{h^{2}}={\left(\Gamma_{22}^{1}\right)}_{i,j}^{n}\frac{f_{i+1,j}^{n}-f_{i-1,j}^{n}}{2h}+{\left(\Gamma_{22}^{2}\right)}_{i,j}^{n}\frac{f_{i,j+1}^{n}-f_{i,j-1}^{n}}{2h}+\frac{N_{i,j}^{n}}{\sqrt{E_{i,j}^{n}+G_{i,j}^{n}-1}}\\ \frac{f_{i+1,j}^{n+1}-f_{i+1,j-1}^{n+1}-f_{i-1,j+1}^{n+1}-2f_{i,j}^{n+1}+f_{i,j-1}^{n+1}+f_{i,j+1}^{n+1}+f_{i-1,j}^{n+1}}{2h^{2}}={\left(\Gamma_{12}^{1}\right)}_{i,j}^{n}\frac{f_{i+1,j}^{n}-f_{i-1,j}^{n}}{2h}+{\left(\Gamma_{12}^{2}\right)}_{i,j}^{n}\frac{f_{i,j+1}^{n}-f_{i,j-1}^{n}}{2h}+\frac{M_{i,j}^{n}}{\sqrt{E_{i,j}^{n}+G_{i,j}^{n}-1}} \end{array}\right.$$

where

$${\left(\Gamma_{11}^{1}\right)}_{i,j}^{n}=\frac{G_{i,j}^{n}(E_{i+1,j}^{n}-E_{i-1,j}^{n})-2F_{i,j}^{n}(F_{i+1,j}^{n}-F_{i-1,j}^{n})+F_{i,j}^{n}(E_{i,j+1}^{n}-E_{i,j-1}^{n})}{4(E_{i,j}^{n}G_{i,j}^{n}-{\left(F_{i,j}^{n}\right)}^{2})h},$$

$${\left(\Gamma_{11}^{2}\right)}_{i,j}^{n}=\frac{2E_{i,j}^{n}(F_{i+1,j}^{n}-F_{i-1,j}^{n})-E_{i,j}^{n}(E_{i,j+1}^{n}-E_{i,j-1}^{n})-F_{i,j}^{n}(E_{i,j+1}^{n}-E_{i,j-1}^{n})}{4(E_{i,j}^{n}G_{i,j}^{n}-{\left(F_{i,j}^{n}\right)}^{2})h},$$

$${\left(\Gamma_{22}^{1}\right)}_{i,j}^{n}=\frac{2G_{i,j}^{n}(F_{i,j+1}^{n}-F_{i,j-1}^{n})-G_{i,j}^{n}(G_{i+1,j}^{n}-G_{i-1,j}^{n})-F_{i,j}^{n}(G_{i,j+1}^{n}-G_{i,j-1}^{n})}{4(E_{i,j}^{n}G_{i,j}^{n}-{\left(F_{i,j}^{n}\right)}^{2})h},$$

$${\left(\Gamma_{22}^{2}\right)}_{i,j}^{n}=\frac{E_{i,j}^{n}(G_{i,j+1}^{n}-G_{i,j-1}^{n})-2F_{i,j}^{n}(F_{i,j+1}^{n}-F_{i,j-1}^{n})+F_{i,j}^{n}(G_{i+1,j}^{n}-G_{i-1,j}^{n})}{4(E_{i,j}^{n}G_{i,j}^{n}-{\left(F_{i,j}^{n}\right)}^{2})h}$$

$${\left(\Gamma_{12}^{1}\right)}_{i,j}^{n}=\frac{G_{i,j}^{n}(E_{i+1,j}^{n}-E_{i-1,j}^{n})-F_{i,j}^{n}(G_{i+1,j}^{n}-G_{i-1,j}^{n})}{4(E_{i,j}^{n}G_{i,j}^{n}-{\left(F_{i,j}^{n}\right)}^{2})h},$$

and

$${\left(\Gamma_{12}^{2}\right)}_{i,j}^{n}=\frac{E_{i,j}^{n}(G_{i+1,j}^{n}-G_{i-1,j}^{n})-F_{i,j}^{n}(E_{i,j+1}^{n}-E_{i,j-1}^{n})}{4(E_{i,j}^{n}G_{i,j}^{n}-{\left(F_{i,j}^{n}\right)}^{2})h}.$$

Constraint conditions are added to Gaussian Equation (2) to guarantee that the simulated value at the lth sampled location $\left(x_{i,}y_{j}\right)$ in the calculation domain is equal to or approximates the observation ${\bar{f}}_{i,j}$ in the corresponding grid. Therefore, the mathematical formula of HASM is given by

(3) $$\left\{\begin{array}{c} \min{∥\left[\begin{array}{c} A\\ B\\ C \end{array}\right]z^{n+1}-{\left[\begin{array}{c} d\\ q\\ p \end{array}\right]}^{n}∥}_{2}\\ s.t.Sz^{\left(n+1\right)}=k \end{array}\right.$$

where each element of the vector z denotes the estimated value of the grid; the constraint equation Sz=k indicates that the predictor estimation is equal to the observation at each station location. If there are m stations in the computational region, the matrix S can be given as

$$S={\left[\begin{array}{cccccccccc} 0 & \cdots & 0 & 0 & 1 & 0 & \cdots & 0 & \cdots & 0\\ 0 & 1 & 0 & 0 & \cdots & \cdots & \cdots & 0 & \cdots & 0\\ \vdots & \vdots & \vdots & \vdots & \vdots & \vdots & \vdots & \vdots & \vdots & \vdots \\ 0 & \cdots & 0 & 0 & 0 & \cdots & 0 & 1 & \cdots & 0 \end{array}\right]}_{m\times (M\times N)}$$

where M=I+2 and N=J+2 are the grid numbers in the x and y directions, respectively. The number of rows in the matrix S is equal to the station number m. If the lth station has a DO concentration of ${\bar{f}}_{i,j}$ and is located in the ith row and jth column in the computational grid, then S(l,(i−1)⋅N+j)=1, $k(l)={\bar{f}}_{i,j}$.

By applying the Lagrange multiplier method, the HASM Equation (3) can be written as

(4) $$\bar{A}z^{n+1}={\bar{b}}^{n}$$

where $\bar{A}=A^{T}A+B^{T}B+C^{T}C+\lambda^{2}S^{T}S$ is a symmetric and positive definite matrix and $\bar{b}=A^{T}d+B^{T}q+C^{T}p+\lambda^{2}S^{T}k$. The Lagrange parameter λ is the weight of the observations. A small value of λ is given in areas with large DO variations. By setting the initial value $z^{\left(0\right)}$ and constructing the constraint equation Sz=k using station observations, the surface modeling of climate variables can be performed by HASM.

### 3.2. HASM_MOD

A disadvantage of HASM is the lack of consideration of the background information associated with predictors while only considering the station observations and their spatial autocorrelation. In this section, a HASM-based method was developed to estimate the spatial distribution of DO by introducing a drift term, which integrates the explanatory variables by using geographically weighted regression (GWR) in the model. The main equation of HASM_MOD is as follows:

(5) $$\underset{z}{\min}{\left\|\bar{A}z-\bar{b}\right\|}_{2}+\beta {\left\|z-\overset{⌢}{z}\right\|}_{2}$$

From a mathematics perspective, the term ${\left\|z-\overset{⌢}{z}\right\|}_{2}$ can be seen as a regularization penalty term for ${\left\|\bar{A}z-\bar{b}\right\|}_{2}$ and can be referred to as L2 regularization. However, L1 penalty methods often outperform L2 penalty methods, especially when irrelevant features are present in $\overset{⌢}{z}$ [50]. Therefore, we use L1 regularization in this study. Replacing the drift term in (5) with the L1 norm yields the following expression:

(6) $$\underset{z}{\min}{\left\|\bar{A}z-\bar{b}\right\|}_{2}+\beta {\left\|z-\overset{⌢}{z}\right\|}_{1}$$

where $\overset{⌢}{z}=GWR(DO,x_{1},x_{2},\cdots ,x_{n})$; $x_{1},x_{2},\cdots ,x_{n}$ represent the auxiliary variables that are correlated with DO levels, such as water temperature and pH; z is an estimate of DO; and β is a regularization parameter. The split Bregman iteration was used to solve the optimization problem (6) [51,52]. The solution process is given as follows:

For $H(z)={\left\|\bar{A}z-\bar{b}\right\|}_{2}$ and $\Phi (z)=|z-\overset{⌢}{z}|$, formula (6) can be rewritten as

(7) $$\underset{z}{\min}H(z)+\beta \Phi (z)$$

Additionally, with d=Φ(z), problem (7) can be converted to

(8) $$\underset{z,d}{\min}H(z)+\beta |d|,s.t.\;d=\Phi (z))$$

Then, this problem can be modified to obtain

(9) $$\underset{u,d}{\arg\min}H(u)+\beta |d|+\frac{\alpha }{2}{\left\|d-\Phi (z)\right\|}_{2}^{2}$$

The optimization Equation (9) can be solved by using the following alternating iterative method:

$$\left\{\begin{array}{c} z^{\left(n+1\right)}=\underset{z}{\arg\min}H(z)+\frac{\alpha }{2}{\left\|d^{\left(n\right)}-\Phi (z)-b^{\left(n\right)}\right\|}_{2}^{2}\\ d^{\left(n+1\right)}=\underset{d}{\arg\min}\beta |d|+\frac{\alpha }{2}{\left\|d-\Phi (z^{\left(n+1\right)})-b^{\left(n\right)}\right\|}_{2}^{2}\\ b^{\left(n+1\right)}=b^{\left(n\right)}+\Phi (z^{\left(n+1\right)})-d^{\left(n+1\right)} \end{array}\right.$$

where z can be resolved by using the Gauss–Seidel algorithm and d can be resolved by

(10) $$d^{\left(n+1\right)}=\mathrm{shrink}(\Phi (z^{\left(n+1\right)})+b^{\left(n\right)},\frac{1}{\alpha })$$

where

$$\mathrm{shrink}(x,y)=\frac{x}{\left\|x\right\|}\cdot \max(\left\|x\right\|-y,0).$$

The optimal value of α can be obtained through sensitivity experiments, and β can be optimized by using the L-curve method [53]. The z result is the final estimate of the DO concentration.

### 3.3. Support Vector Machine

The SVM method, which is developed based on statistical learning theory, has been successfully applied to classification and regression problems. The basic concept behind SVM is to map the original datasets to higher-dimensional features of space and construct an optimal separating plane (SP), from which the distance to all the data points is minimal [54]. For training data set $\left\{(x_{i},y_{i}),i=1,\cdots ,n\right\}$,$x\in R^{m}$,y∈R, where n is the total number of data patterns, x is the input vector of m components, and y is the corresponding output value, the SVM regression function can be expressed as follows:

(11) f(x)=θ⋅ϕ(x)+e

where θ is the weight vector, e is the bias, and ϕ(x) indicates the nonlinear transfer function. The parameters θ and e, which define the location of SP, can be determined by minimizing the following regularized risk function:

(12) $$\min\frac{1}{2}{\left\|\theta \right\|}^{2}+\beta \sum_{i=1}^{n}(\xi_{i}+\xi_{i}^{*})$$

*subject to* $y_{i}-\theta \cdot \phi (x)-e\leq \varepsilon +\xi_{i}\;\theta \cdot \phi (x)+e-y_{i}\leq \varepsilon +\xi_{i}{}^{*},\xi_{i}\geq 0,\xi_{i}{}^{*}\geq 0$, where β is the regularization parameter, $\xi_{i}$ and $\xi_{i}{}^{*}$ are slack variables, and problem (12) is solved in a dual form using the Lagrangian multipliers.

(13) $$\max-\frac{1}{2}\sum_{i=1}^{n}\sum_{j=1}^{n}(c_{i}-c_{i}^{*})(c_{j}-c_{j}^{*})K(x_{i},x_{j})-\sum_{i=1}^{n}(c_{i}-c_{i}^{*})+\sum_{i=1}^{n}(c_{i}-c_{i}^{*})y_{i}$$

*subject to* $\sum_{i=1}^{n}(c_{i}-c_{i}^{*})$, $0\leq c_{i}\leq C,0\leq c_{i}^{*}\leq C$. $K(x_{i},x_{j})$ *is the kernel function*.

By imposing the Karush–Kuhn–Tucker (KKT) optimality condition, $\theta^{*}$ is obtained, that is,

(14) $$\theta^{*}=\sum_{i=1}^{n}(c_{i}-c_{i}^{*})\cdot K(x_{i},x)$$

Finally, the SVM is expressed as follows:

(15) $$f(x)=\sum_{i=1}^{n}(c_{i}-c_{i}^{*})\cdot K(x_{i},x)+e$$

The radial basis function (RBF) was adopted as the kernel function of SVM in this study [55].

### 3.4. Cokriging Method

Cokriging is the multivariate equivalent to kriging. The general form of the kriging equation is

(16) $$z^{*}(x_{p})=\sum_{i=1}^{n}\lambda_{i}z(x_{i})$$

In order to achieve unbiased estimations in kriging, the following set of equations should be solved:

(17) $$\left\{\begin{array}{l} \sum_{i=1}^{n}\lambda_{i}\gamma (x_{i},x_{j})-\mu =\gamma (x_{i},x)\\ \sum_{i=1}^{n}\lambda_{i}=1 \end{array}\right.$$

where $z^{*}(x_{p})$ is the estimated value at location $x_{p}$, $z(x_{i})$ is the known value at location $x_{i}$, $\lambda_{i}$ is the weight associated with the data, μ is the Lagrange coefficient, and $\gamma (x_{i},x_{j})$ is the value of variogram corresponding to a vector with origin in $x_{i}$ and extremity in $x_{j}$.

By using multiple datasets, cokriging is a very flexible interpolation method, allowing the user to investigate graphs of cross-correlation and autocorrelation [17]. The general equations of cokriging estimator are [18]

(18) $$\left\{\begin{array}{l} \sum_{l=1}^{v}\sum_{i=1}^{n}\lambda_{il}\gamma_{lv}(x_{i},x_{j})-\mu_{v}=\gamma_{uv}(x_{j},x)\\ \sum_{i=1}^{nl}\lambda_{il}=\left\{\begin{array}{ll} 1, & 1=u\\ 0, & 1\neq u \end{array}\right. \end{array}\right.$$

where u and v are the primary and covariate variables, respectively.

In the cokriging method, the u and v variables are cross-correlated, and the covariate contributes to the estimation of the primary variable. For cokriging analysis, the cross variogram should be determined beforehand. Provided that there are points where both u and v have been measured, the semivariogram can be estimated by [18]

(19) $$\gamma_{uv}(h)=2\frac{1}{N(h)}\sum_{i=1}^{N(h)}\left\{z_{u}(x_{i})-z_{u}(x_{i}+h)\right\}\left\{z_{v}(x_{i})-z_{v}(x_{i}+h)\right\}$$

The cokriging was implemented by using the “Geostatistical Analyst” tool in ArcGIS software, which can automatically optimize the parameters based on the input datasets.

### 3.5. Performance Assessment of the Methods

In this study, the performance of HASM_MOD was assessed by using a cross-validation method, namely the leave-one-out cross-validation, in which each observation was considered as the validation set and the remaining observations were considered as the training set [56]. Three commonly used evaluation indicators, namely the mean absolute error (MAE), root-mean-square error (RMSE), and coefficient of determination ($R^{2}$), were used to compare the performance of different methods. MAE can accurately reflect the actual simulation error, as it can measure the difference between simulations and observations. The RMSE indicates the fit of the method to the observed data. A high value indicates that large deviations exist between the fitted values and the observations. $R^{2}$ reflects how well the model fits the observed data and typically gives the percentage of the variation in a variable that can be explained by the model.

$$R^{2}=(\frac{\sum_{i=1}^{N}\left(p_{i}-\bar{p})(o_{i}-\bar{o}\right)}{\sqrt{\sum_{i=1}^{N}{\left(p_{i}-\bar{p}\right)}^{2}\sum_{i=1}^{N}{\left(o_{i}-\bar{o}\right)}^{2}}}{)}^{2}$$

$$MAE=\frac{1}{N}|p_{i}-o_{i}|$$

$$RMSE=\sqrt{\frac{1}{N}\sum_{i=1,\cdots ,N}{\left(p_{i}-o_{i}\right)}^{2}}$$

where $o_{i}$ is the observation value at the ith point $\left(x_{i},y_{i}\right)$, $p_{i}$ is the estimate, $\bar{p}=\frac{1}{N}\sum_{i=1}^{N}p_{i}$, $\bar{o}=\frac{1}{N}\sum_{i=1}^{N}o_{i}$, and N is the number of test points.

## 4. Results

The relationships between the candidate factors and the DO level were investigated by using Spearman’s rank method with a significance level of 1%. In this study, we first took July and August in the wet season as examples. In July, the candidate inputs, namely WT, PH, Chl-a, and Pre, were significantly correlated with DO, and the corresponding Spearman rho values were −0.58, 0.45, 0.52, and 0.83, respectively. In August, the candidate factors WT, Chl-a, AP, and Pre were strongly correlated with DO, and the corresponding Spearman rho values were −0.64, 0.47, 0.57, and 0.52, respectively. Finally, according to the results of Spearman’s rank method among the candidate factors, the explanatory variables related to the DO concentration in July included WT, PH, Chl-a, and Pre, and the most relevant environmental variables for DO in August included WT, Chl-a, AP, and Pre.

A comparison of the simulated and observed values based on cross-validation is shown in Table 1; notably, HASM_MOD performs the best in July and August. The MAEs of the prediction results of HASM_MOD, HASM, the SVM, and cokriging are 0.14, 0.22, 0.27 and 0.39, respectively, in July, and 0.22, 0.30, 0.41, and 0.30, respectively, in August. Thus, according to the MAE, HASM_MOD performs best, followed by HASM in July; however, in August, cokriging performs better than HASM according to the MAE and RMSE. The RMSE values for HASM_MOD were 28%, 46%, and 42% lower than those of HASM, the SVM, and cokriging in July and August on average. From the fitting effect (R^2^), HASM_MOD gives the best results, followed by HASM, while the performance of cokriging and the SVM varies in different months.

**Figure 3:**
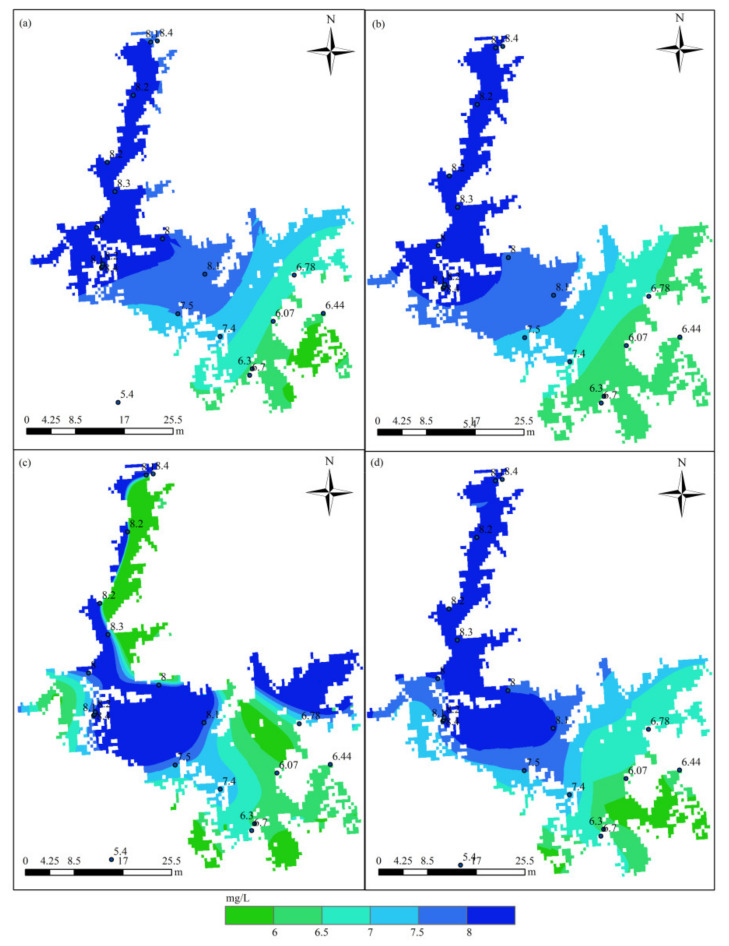
Spatial distribution of DO in Poyang Lake in July using different methods: (**a**) cokriging; (**b**) the SVM; (**c**) HASM; (**d**) HASM_MOD.

**Figure 4:**
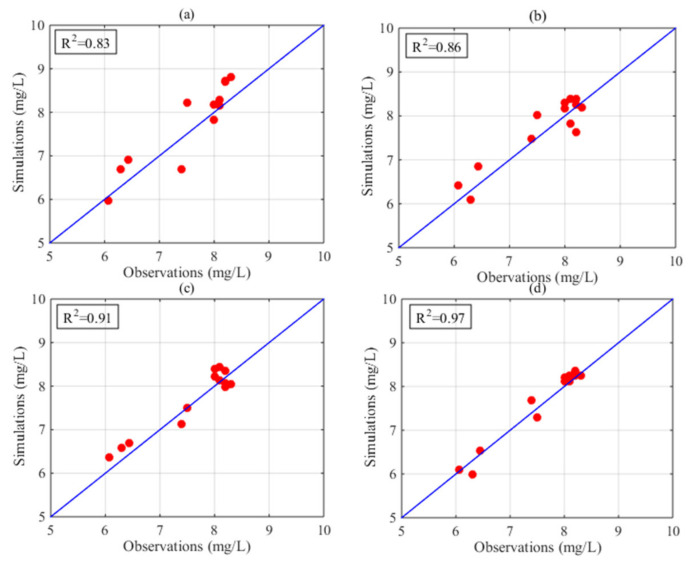
Observed and estimated DO in July from (**a**) cokriging, (**b**) the SVM, (**c**) HASM, and (**d**) HASM_MOD.

Figure 3 displays the distribution of DO in Poyang Lake in July based on the HASM_MOD, HASM, cokriging, and SVM methods. The HASM results display localized patterns with large DO concentrations surrounded by low values in the lake. The HASM_MOD, cokriging, and SVM results exhibit similar patterns, with large values found in the north and low values in the southeast. Although HASM_MOD, cokriging, and the SVM yield similar patterns, large differences exist in some local areas, such as in the middle of the lake. Compared with the observations obtained from 18 stations located in the lake and along the surrounding boundary, the results of HASM_MOD were better than those of cokriging and the SVM (Figure 3a,b,d); cokriging and the SVM tended to underestimate the DO concentrations, especially in the southern part of the lake. The HASM results seem to generally fit the actual observations, but there are some oscillations, especially near the station locations; these variations may be due to the equation systems used in HASM. Furthermore, with data from 11 stations located in the lake, the results of different methods were validated through cross-validation. The scatter correlation plots of the observed and simulated values in July (Figure 4) also suggest that HASM_MOD estimated DO concentrations reliably, with an R^2^ of 0.97; this value was 7%, 13%, and 17% higher than those for HASM, the SVM, and cokriging, respectively.

**Figure 5:**
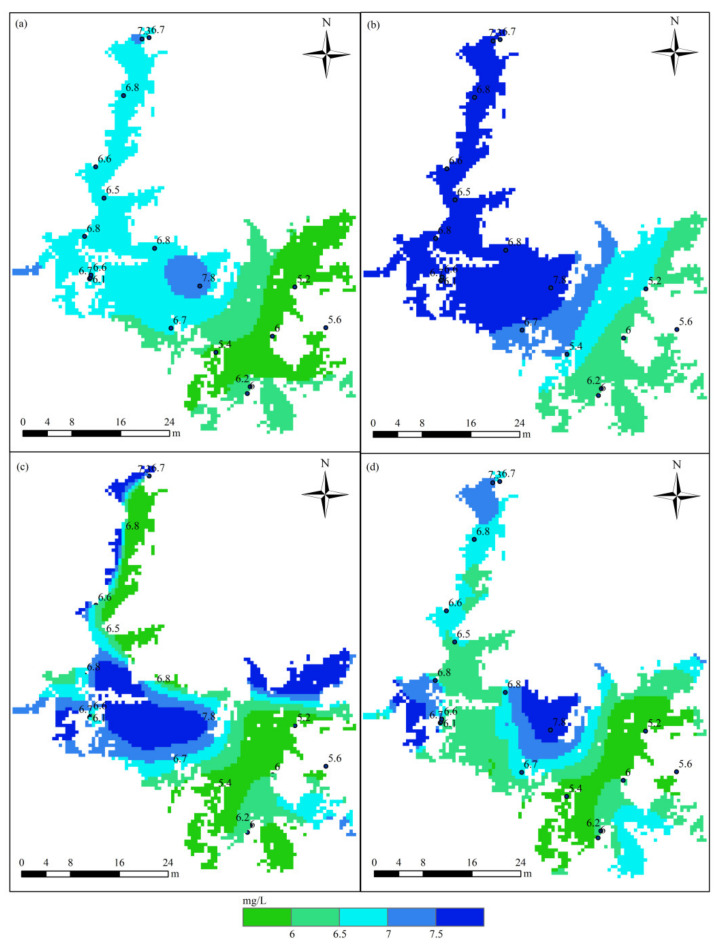
Spatial distribution of DO in Poyang Lake in August using different methods: (**a**) cokriging; (**b**) the SVM; (**c**) HASM; (**d**) HASM_MOD.

**Figure 6:**
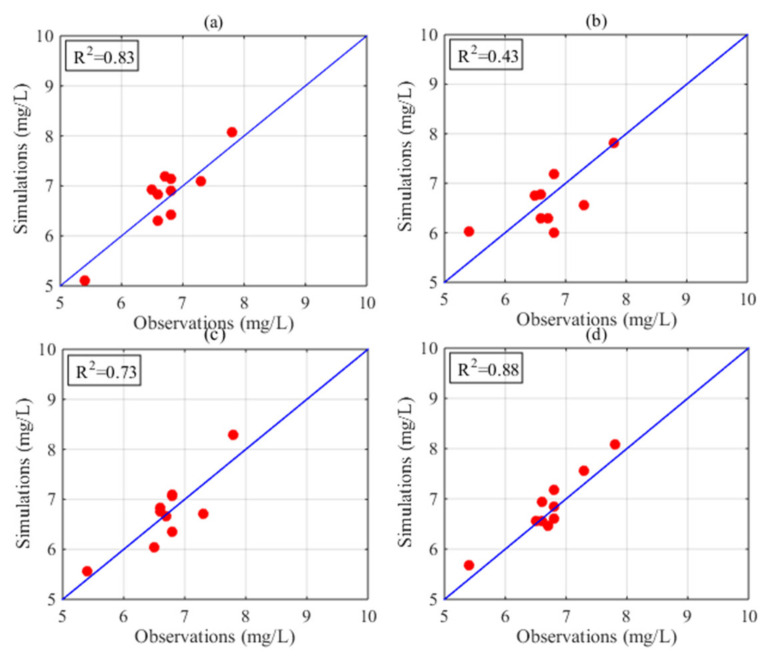
Observed and estimated DO in August from (**a**) cokriging, (**b**) the SVM, (**c**) HASM, and (**d**) HASM_MOD.

The performance of the methods in August is displayed in Figure 5. Obvious differences existed among the spatial patterns of DO concentrations resulting from different methods. Compared with the 18 station observations, the results of HASM_MOD were generally best. Cokriging tended to underestimate the actual values in some local areas, and the SVM exhibited overestimations in most parts of the lake (Figure 5a,b). Compared with HASM, HASM_MOD performed better at station locations (Figure 5c,d). Figure 6 illustrates the relationship between the observations of 10 stations located in the lake and the corresponding estimated values. The SVM performed the worst, with an R^2^ of 0.43, and the HASM_MOD results displayed the best agreement with site observations (Figure 6d), followed by cokriging, with an R^2^ of 0.83.

**Figure 7:**
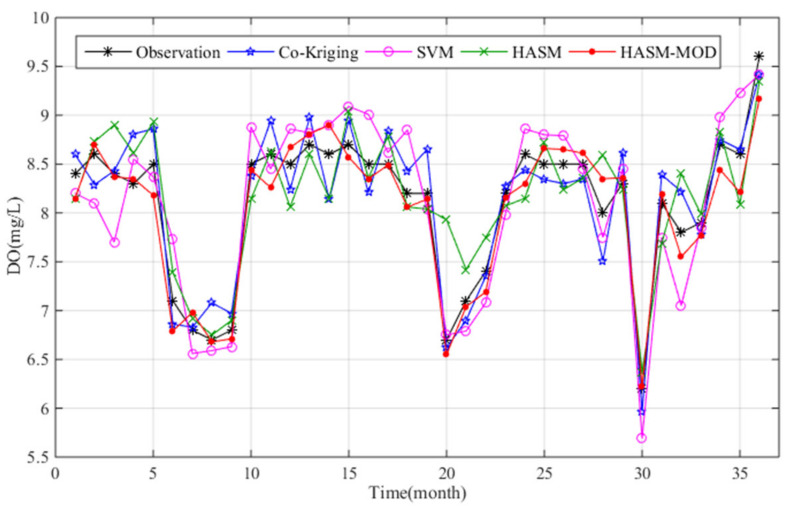
Comparison of the results of different methods with the observations at Sanshan station from January 2015 to December 2017.

We also compared the accuracy of the estimated values and real observations at two stations from January 2015 to December 2017. The sites selected were the Sanshan station located in the middle of the lake and the Outlet_A station located at the outlet of Poyang Lake. Figure 7 shows the simulated DO concentrations at Sanshan station by using the cross-validation method. The predictions obtained from HASM_MOD are closer to the observations than the estimates of the other three methods and have lower prediction errors (Table 2). Large biases between the simulated and observed values were found for HASM and the SVM (Figure 7), with RMSE values of 0.37 and 0.36, respectively. HASM_MOD performed better than cokriging, the SVM, and HASM, with MAE reductions of 25%, 42%, and 38%, respectively. The SVM produced the worst results according to the MAE, and HASM yielded the worst results in terms of the RMSE and R^2^, indicating that HASM tends to produce outliers.

**Figure 8:**
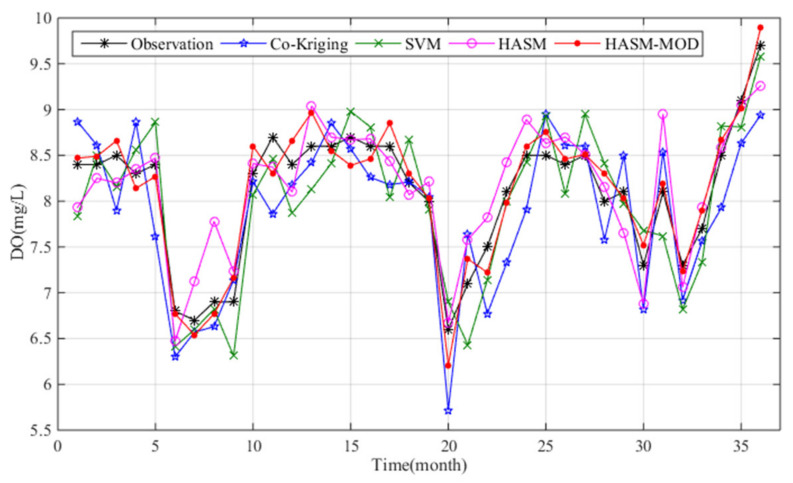
Comparison of the results of different methods with the observations at the Outlet_A station from January 2015 to December 2017.

The monthly averages versus observed values at the Outlet_A station from 2015–2017 are shown in Figure 8. Large biases between the observations and estimates can be observed for HASM and cokriging, with maximum biases of 0.89 and 0.88 mg/L, respectively. Cokriging performed the worst; the SVM outperformed HASM based on the RMSE and R^2^, and HASM performed better than the SVM according to the MAE. HASM_MOD performed the best, with the MAE of 0.17 mg/L, RMSE of 0.20 mg/L, and R^2^ of 0.93 (Table 2). According to the MAE, the accuracy of HASM_MOD was 37%, 48%, and 59% higher than the accuracies of HASM, the SVM, and cokriging, respectively.

## 5. Discussion

To obtain highly accurate estimates of DO in the lake, a model based on HASM was proposed in this study. Previous studies have indicated that a PDE-based method could be effective in reducing the uncertainty in environmental variable simulations [28,57]. A high-accuracy surface modeling (HASM) method in terms of the differential geometry of surfaces was recently proposed, and studies have demonstrated that HASM yields good performance in simulating some environmental variables [29,30,31,32,33]. To obtain improved estimates of DO concentrations, HASM was extended in this study.

The results show that HASM produced lower MAE values and higher RMSE and R^2^ values than SVM and cokriging, indicating that some outliers may exist in the HASM results. The constraint equations in HASM based on station observations result in low MAE values; therefore, the HASM-simulated values are approximately equal to the real measurements at station locations. Furthermore, due to the restrictions of the constraint equations and equation (2), which consider the spatial autocorrelation of DO by using a finite difference scheme, the spatial patterns of DO produced by HASM oscillate, with generally accurate simulated values near station locations. The contributions of these biases of HASM mainly lie in the input datasets and the method of solving the differential equations (1). Although HASM takes into account the spatial autocorrelation of DO by using finite difference schemes, it ignores the correlation between the DO and the related explanatory variables. To eliminate the oscillation phenomenon and obtain high-accuracy DO estimates, a regularization penalty term obtained by using the local regression method together with some auxiliary variables related to DO was introduced in HASM; the resulting model was called HASM_MOD. The local regression method was implemented by using GWR, which enables the relationship between the DO concentration and the corresponding explanatory variables to vary by region. HASM_MOD integrates station observations by using HASM and the explanatory variables using GWR and can produce improved results. The results indicate that HASM_MOD is a reliable method for DO concentration prediction. The accuracy of the HASM_MOD results was not influenced by the characteristics of the time series. For the nonstationary time series of monthly mean DO concentrations at the Sanshan and Outlet_A stations, HASM_MOD yields satisfying results, whereas HASM, the SVM, and cokriging generate unreliable results, as demonstrated by the large RMSE values. The errors of HASM_MOD were reduced due to the regularization penalty term, which takes into account the spatial heterogeneity by integrating the explanatory variables using GWR. Although some researchers showed that SVMs can well predict the dissolved oxygen in aquaculture [58], the performance of SVMs was unsatisfactory in estimating the DO concentration in Poyang Lake. This may be due to the non-optimum parameters of SVMs and the kernel function used in this study, and studies have indicated that there is no effective method to obtain the optimal parameter combination of SVMs accurately [38]. The accuracy of cokriging was also not satisfactory according to the cross-validation process, which may be due to its linear nature and the failure to take into account the spatial nonstationarity of DO concentration. Compared with that of the other three methods, the performance of HASM_MOD was not influenced by the spatial distribution of the DO concentration. The good performance of HASM_MOD may be due to the combined effect of the constraint equations in HASM and the use of auxiliary variables in GWR, which integrates the spatial autocorrelation of DO concentrations; the characteristics of spatial variation of the DO concentrations, such as the direction and slope of the variation; and the local correlation between the DO concentrations and the related explanatory variables. The results indicate that the accuracy of HASM_MOD was improved by 38%, 45%, and 42% compared with the accuracies of HASM, the SVM, and cokriging, respectively.

With the correct selection of explanatory variables, it is possible to accurately estimate the DO concentration in Poyang Lake by using HASM_MOD. The explanatory variables vary with time scale, and in this study, we used Spearman’s rank correlation test to select the auxiliary variables related to DO at a significance level of 1%. One of the obstacles is the difficulty in finding the appropriate spatial and temporal scientific datasets for target areas. Further studies can be conducted by introducing other explanatory variables and applying remote sensing techniques. HASM_MOD is expected to yield good performance in other areas at hourly scales, which will be investigated further in the future.

## 6. Conclusions

In this research, a model was built to yield highly accurate DO estimates by integrating station observations and explanatory variables using HASM and GWR. The developed model comprehensively considers the spatial autocorrelation of DO and correlations with other environmental variables based on station observation constraints in terms of the fundamental theorem of surfaces. By applying a cross-validation method, HASM_MOD improved the accuracy of prediction, with MAE reductions of 38%, 45%, and 42% compared with HASM, SVM, and cokriging, respectively. The proposed method provides a new way to estimate DO concentrations with high accuracy, and this method could be successfully applied to estimate other water quality parameters. Further work will consider additional explanatory variables and remote sensing techniques and focus on improving predictions of DO levels at the hourly scale.

## Figures and Tables

**Table 1:** Modeling evaluation indices using the cross-validation method.

Month	July			August		
Error	MAE	RMSE	R^2^	MAE	RMSE	R^2^
Cokriging	0.39	0.45	0.93	0.30	0.32	0.96
SVM	0.27	0.31	0.96	0.41	0.47	0.83
HASM	0.22	0.24	0.98	0.30	0.34	0.97
HASM_MOD	0.14	0.17	0.99	0.22	0.25	0.97

**Table 2:** Errors obtained with different methods.

Station	Outlet A			Sanshan		
Error	MAE	RMSE	R^2^	MAE	RMSE	R^2^
Cokriging	0.41	0.47	0.72	0.24	0.27	0.88
SVM	0.33	0.34	0.82	0.31	0.36	0.86
HASM	0.27	0.37	0.78	0.29	0.37	0.77
HASM_MOD	0.17	0.20	0.93	0.18	0.20	0.93

## Data Availability

The data are not publicly available due to the confidentiality of the research projects.
